# Supplementary material for: Imperfect language learning reduces morphological overspecification: Experimental evidence
Source: PLoS One. 2022 Jan 27;17(1):e0262876. doi: 10.1371/journal.pone.0262876 (PMC8794192; doi:10.1371/journal.pone.0262876)
Supplement: S1 Appendix — (ZIP) [file pone.0262876.s001.zip › Appendix S1/readme.docx]

#### Appendix S1. Dataset; detailed results; code

data_analyzer.rb is a Ruby script which calculates all the measures discussed in the article. See comments within the file for more information. Ruby 1.9.3 or higher is required to run the script (install Ruby, and run ruby analyzer.rb from the command line).

The folder Measures contains the output of data_analyzer.rb (a separate csv file for every measure and every condition, files v364*.csv).

The folder Languages contains all the 495 languages (files lang*.csv) and basic data (file rough_data2.csv) about the participants. data_analyzer.rb uses these data as input.

statistics.r is an R script which performs the statistical analysis described in section 4. The comments in the scripts explain, *inter alia*, how the models were selected and how the assumptions were tested.

epsilon-graphs.R is an R script which makes the line graphs and the boxplot figures in the article.

The archive clean_code.zip contains the full code for the webpage where the experiment was hosted.

CausalLinksEpsilon.csv is a machine-readable representation of the causal graph (Fig. 8 in the main text).
