# Supplementary material for: Imperfect language learning reduces morphological overspecification: Experimental evidence
Source: PLoS One. 2022 Jan 27;17(1):e0262876. doi: 10.1371/journal.pone.0262876 (PMC8794192; doi:10.1371/journal.pone.0262876)
Supplement: S1 Text — (DOCX) [file pone.0262876.s002.docx]

#### Text S1. Instructions to the participants

The original instructions were in Russian, we provide English translations here. For brevity’s sake we omit some less important messages and technical details.

*Introductory instructions 1*

PLEASE READ THE TEXT BELOW CAREFULLY

The goal of the experiment is to test some current linguistic hypotheses. It will take **10-20** minutes, during which you will try to learn a language you do not know, that of the planet Epsilon. If you are less than 16 years old, or if the Russian language is not your native language, or if you are a linguist, we kindly ask you to refrain from participating (all of these factors can influence your results, and we would like to know how an average adult Russian speaker behaves in these conditions).

The principal investigator is Aleksandrs Berdicevskis, UiT The Arctic University of Norway. The experiment is conducted in accordance with the Norwegian law. Your participation is voluntary, you can stop the experiment at any time. Your participation is anonymous: we are not collecting or storing names, email addresses, IP-addresses or other data that would allow us to identify the participants. Other data (age, gender, results) will be permanently stored by the researchers and can later be used for analysis and publication. Prize codes (see next page) are stored separately from all of the other data and cannot be matched against them, after handing out the prizes all of the codes will be destroyed.

**Please press the spacebar to continue.**

*Introductory instructions 2*

**Rules of the experiment**

Before starting the experiment, please make sure that you can spend the next 20 minutes solely on the experiment without being distracted by anything else (talking, music, email, chat). **Do not write anything down,** neither on a piece of paper, nor on a computer (except when asked to type in an answer). Do not participate in the experiment more than once.

We cannot control that you follow these rules, but we rely on your honesty. Breaking the rules might lead to a drastic distortion of our results. If you for some reason did not follow the rules, please let us know at [aleksandrs.berdicevskis@uit.no](mailto:aleksandrs.berdicevskis@uit.no).

**Reward**

The participants who finish the experiment will take part in a raffle. The winners will get books of their choosing from an Internet store (Amazon, Ozon, Labyrinth or any other legally working store where we will be able to make a purchase) for a sum of up to 70 euros (including delivery), we will pay for the order.

At the end of the experiment, you will get several codes. Each code is one of your raffle tickets. The better you perform in the experiment, the more codes you get, and the higher your chances to win are! Save all of the codes in their entirety. At the end of the experiment one code will be randomly chosen, and a part of the code will be published on this website. In order to get the prize you will need to contact us at the email address above and send us the second part of the code.

**Press the space bar to continue.**

*Experiment instructions*

INSTRUCTIONS

You are participating in a space expedition, establishing contact with the inhabitants of a faraway planet called Epsilon. The Epsilonians are friendly and are happy to show you their planet. Today an Epsilonian named Seusse wants to teach you his language.

He will show you photos of Epsilonian animals and describe them in the Epsilon language. Do not press any keys, just watch. From time to time Seusse will be checking on how you are learning the language. Always answer the questions, even if you are not certain about your answer.

Do not worry if you think that you are not doing well: your friend will put all of his effort into understanding you. The most important thing is for him to see that you are really trying. Do not spend too much time on your answers. If you take too much time, Seusse will tell you. If that happens, please answer as fast as possible, or Seusse will become upset at you. **Do not write anything down and do not get distracted**.

Good luck!

**Ready to start? Press the space bar.**

*Meaning-to-signal task, English translation*

Describe this picture in Epsilon in such a way that Seusse could understand you.

*Signal-to-meaning task, English translation*

Seusse said the following sentence: ‘<sentence>’. What does he mean? Press the corresponding key (1–6) on the keyboard
