# Supplementary material for: Imperfect language learning reduces morphological overspecification: Experimental evidence
Source: PLoS One. 2022 Jan 27;17(1):e0262876. doi: 10.1371/journal.pone.0262876 (PMC8794192; doi:10.1371/journal.pone.0262876)
Supplement: S2 Text — (DOCX) [file pone.0262876.s003.docx]

#### Text S2. Recruiting and filtering participants in an online setting

In all of the trials of the signal-to-meaning, meaning-to-signal and interim training blocks, if the participants did not respond within the first fifteen seconds after the stimulus presentation, they were prompted to answer within the next two minutes, and were told that otherwise their alien Seusse friend would become upset. If the participants did not respond within the next two minutes, they were alerted that Seusse became upset. In these cases the experiment continued after the message without any other messages to the participant, but the participant’s data were not included in the final analysis and another subject was assigned to the same chain and generation afterwards. This filter was implemented in order to make sure that if the participants for some reason lost focus, switched to some other activity and later returned to the experiment with a worse proficiency in the language, these extraexperimental reasons for change in the structure of the language would not affect the results. The participants who were filtered out by this mechanism still got their codes and participated in the prize drawing on a par with others. The number of subjects reported in the article (450) does not include these participants.

On a methodological note, we would like to highlight that our approach to recruitment is different from typical recruitment methods for linguistic experiments in both offline and online settings. In the former, the participants are usually university students. In the latter, they are usually recruited through MTurk or, more rarely, through social networks. We believe that our approach results in smaller skewness of the sample than the offline and social-network recruiting, while at the same time the subjects are possibly more interested and motivated than average MTurk workers. Finally, the lottery approach does not require large expenses, while still providing a material incentive for participants.

In the online setting, it is impossible to check whether participants followed the instructions to the letter. Their performance (see section 3.2), however, suggested that they did: it was neither perfect (indicating that they did not break the prohibition to take notes, see 2.4), nor very poor (meaning that they did try to learn the languages and perform well during the tests).
