# Supplementary material for: Imperfect language learning reduces morphological overspecification: Experimental evidence
Source: PLoS One. 2022 Jan 27;17(1):e0262876. doi: 10.1371/journal.pone.0262876 (PMC8794192; doi:10.1371/journal.pone.0262876)
Supplement: S3 Text — (DOCX) [file pone.0262876.s004.docx]

#### Text S3. The structure of the input languages

In all initial input languages, noun stems had the structure CVC (consonant-vowel-consonant), verb stems C, redundant agent marker V and plural marker C. Roman letters were used. They were randomly drawn from the English alphabet (excluding *q*, *y*, *x*, *w*), with the following restrictions:

— all 12 letters used to form the aforementioned morphemes (6 for noun stems, 3 for verb stems, 2 for agent markers on verbs, 1 for plural marker on nouns) had to be different.

— if one agent marker was “i”, the other one could not be “e”, and vice versa, same applies to “a” and “o” (the reason is that in Russian, the letters within each of these pairs can denote the same sound in certain phonological contexts).

Finally, the languages were manually checked by the authors. If a language contained words that were too similar to frequent Russian words, it was generated anew, until both authors found it satisfactory.

It can be argued that our participants do not even perceive the verbal endings as agent markers (especially since our "noun classes" have one member each), and invent another rule that explains the observed pattern (e.g. some kind of vowel harmony). Even if it happens, it is not a major obstacle for our purposes. The verbal endings and their distribution remains an instance of overspecification which may or may not disappear.
