# Supplementary material for: Imperfect language learning reduces morphological overspecification: Experimental evidence
Source: PLoS One. 2022 Jan 27;17(1):e0262876. doi: 10.1371/journal.pone.0262876 (PMC8794192; doi:10.1371/journal.pone.0262876)
Supplement: S4 Text — (DOCX) [file pone.0262876.s005.docx]

#### Text S4. Comprehension test

During the comprehension test (see section 2.5) the participants were presented with a signal-to-meaning mapping task with all sixteen pictures as targets. In each trial the participants were presented with the target picture, five distractor pictures and the instructions “Seusse said the following sentence: ‘<sentence>’. What does he mean? Press the corresponding key (1–6) on the keyboard” in order to simulate an actual communication event for the participants. The order in which the target pictures appeared was randomized. The distractor pictures were selected from the experimental stimuli by the following algorithm in order to make the choice of the correct answer maximally difficult and prevent participants from making “educated” guesses, relying on superficial knowledge of the language.

In 30% of the trials the five distractor pictures were chosen randomly among all of the pictures having the same agent as the target picture. In the remaining 70% of the cases the distractor pictures were chosen using the following procedure. First, the remaining three pictures having the same agent in the same grammatical number as in the target picture were added to the pool of possible distractor pictures. For example, if the target picture was the one denoted by *segl ro* in Figure 2, pictures corresponding to *segl, segl mo* and *segl bo* were added to the pool. Additionally, a different randomly chosen group of four pictures all sharing the same agent in the same grammatical number was added to the distractor pool, for example in the case of the language in Figure 2 this group could be either *seg*, *seg mo*, *seg bo*, *seg ro* or *fuv*, *fuv mi*, *fuv bi*, *fuv ri* or *fuvl*, *fuvl mi*, *fuvl bi*, *fuvl ri*. After this, five pictures out of the pool were randomly added as distractors.

The two patterns of distractor picture selection were used to prevent the participants from learning the pattern and adjusting to it.
