# Supplementary material for: Imperfect language learning reduces morphological overspecification: Experimental evidence
Source: PLoS One. 2022 Jan 27;17(1):e0262876. doi: 10.1371/journal.pone.0262876 (PMC8794192; doi:10.1371/journal.pone.0262876)
Supplement: S5 Text — (DOCX) [file pone.0262876.s006.docx]

#### Text S5. Comprehension rate and underspecification rate

The first of the two tests administered during the testing stage of the experiment measured how well a participant was able to find the correct meaning given the signal (see S5). Seeing Seusse’s utterance, the participant had to understand what their alien friend had in mind and to choose a corresponding picture.

The test measures two properties of the input language: first, how learnable it is, second, how expressive it is. It often happens that the input language is underspecified, or, in other words, contains ambiguity: say, the two meanings 'round animal' and 'round animals' both correspond to the same signal *sig* (see language N7-10). In this case, when seeing *sig* during the comprehension test, the participant has to guess which of the two meanings Seusse has in mind, there are no cues that could help to make a choice. This reflects the obvious fact that underspecification in such circumstances is harmful for communication (Kirby et al. 2015). While there is no real communication task in our setting and thus no pressure for expressivity, the goal of framing the comprehension test as a dialogue with Seusse was exactly to create the impression of communication and inhibit the possible trend towards underspecification.

The success rate in the comprehension test is shown on Figure S1. There is a clear decrease in all conditions, but note that the rate never drops below 80%, i.e. is always high.

We hypothesize that there are two reasons for the decrease in comprehension rate. One is that, as discussed in the main text, languages become less regular. Another is that they become more underspecified in the sense that two or more meanings get associated with one stimulus (sentence). To gauge underspecification, we measure how many of the 16 meanings a language cannot unambiguously express. Language N7-10, for instance, can distinguish between 14 meanings ('round animal' is indistinguishable from 'round animals', 'square animal' is indistinguishable from 'square animals'). Thus, two meanings from the 16 available cannot be expressed, and the underspecification is 2/16 = 0.125. Underspecification is shown on Figure S2.

References:

Kirby, S., Tamariz, M., Cornish, H., & Smith, K. (2015). Compression and communication in the cultural evolution of linguistic structure. *Cognition* 141: 87–102. DOI: 10.1016/j.cognition.2015.03.016
