# Supplementary material for: Imperfect language learning reduces morphological overspecification: Experimental evidence
Source: PLoS One. 2022 Jan 27;17(1):e0262876. doi: 10.1371/journal.pone.0262876 (PMC8794192; doi:10.1371/journal.pone.0262876)
Supplement: S6 Text — (DOCX) [file pone.0262876.s007.docx]

#### S6. Further discussion of model validity

Obviously, the model we use is extremely simple, much simpler than the real cognitive and social processes that presumably shape the linguistic structure. In this appendix, we briefly outline some important simplifications that our model relies on, and discuss how they affect its validity.

Some simplifications are *quantitative* in nature. Our "languages" can be said to contain two nouns, three verbs, two morphological rules (expression of number and double agent-marking) and one syntactic rule (noun first, verb second), as opposed to much higher numbers of lexical items and rules in human languages (even the extraordinary cases like pidgins, constructed or emerging languages). Our "generations" consist of one person each, as opposed to hundreds of thousands in the real world (smaller numbers are often indicative of language death). Language learning in the experiment takes 10–20 minutes, while in reality it takes years.

Some simplifications are *qualitative* in nature. There is no communication involved in the task (only a wording designed to instill a feeling of a communication-like situation) and thus no actual use of language. From any usage-based perspective, this is a tremendous simplification, since the language is to a very large extent shaped by use, and, moreover, learning cannot actually be fully separate from use (Beckner et al. 2009). Further, our "normal" learners are not children (nor do we claim them to be) and are not acquiring their *first* language. Obviously, there is no real language contact (our "imperfect" learners did not acquire another artificial language prior to being exposed to Epsilonian).

With these caveats in mind, even the choice of some simplest labels can be disputed: do our participants really learn *languages* that have *grammar*, *sentences* with *meanings* etc.? Or are they just facing the task of memorizing several mappings between images and strings of symbols, which is not equivalent to learning a language?

The answers to these concerns ultimately rely on the assumption that cognitive processes that shape linguistic structure are (mostly) domain-general (Beckner et al. 2009). As toy languages are passed through toy generations, *real* changes occur and have to be explained. One possible (though hardly plausible) explanation is that the changes are just random. Another one is that they are shaped by some cognitive factors specific to the types of learning and production used in the experiment, and thus findings cannot be generalized to conclusions about language. Yet another, the one we find to be most compatible with our current knowledge about language and cognition, is that the cognitive factors at play are (more or less) the same for toy and real languages. While memorizing string–image matches might not be exactly the same as learning a language, the cognitive mechanisms involved might be similar enough to make the model meaningful. Importantly, there exists neuroimaging evidence strongly supporting this assumption (Christiansen, Conway and Onnis 2007; Friederici, Steinhauer and Pfeifer 2002), as well as research suggesting that performance in artificial language learning experiments is correlated with multiple measures of L2 learning ability, even after controlling for IQ (Ettlinger, Morgan-Short, Faretta-Stutenberg, & Wonge, 2016).

That said, the model is still simplistic. This results from both practical and theoretical needs. Experiments with human subjects are difficult and costly. Online settings like the one we use reduce the costs greatly, but require very simple design to keep the participants' attention throughout the task. Moreover, it can be methodologically useful to start with a simple model and test later whether more sophisticated ones provide more realistic output. Not to mention that simplicity can be beneficial, since it makes it possible to focus exclusively on some (presumably crucial) factors (learning in this case).

Finally, an important question is how the validity of models like ours can be estimated. Obviously, we want the changes that occur in the languages to resemble the changes typical for real languages. The problem is, we do not know for sure whether the presence of non-native speakers should lead to simplification (though we have very good reasons to believe it should), and we do not know exactly what this simplification should look like. As a result, the experiment is simultaneously a test of a theoretical prediction by means of a model and an attempt to validate the model. To the best of our knowledge, this problem concerns all iterated learning experiments, and arguably many other modeling approaches. However, even with this caveat in mind, the modeling is still informative: *converging* evidence supports the theory (as in our case), *diverging* evidence shows that the model is not in agreement with the theory (though it might be far from obvious which of the two is wrong and why). Hopefully, if our understanding of the iterated learning models themselves grows with experience, so will our ability to tailor the most valid models.

References:

Beckner, C., Blythe, R., Bybee, J., Christiansen, M., Croft, W., Ellis, N.C., Holland, J., Ke, J., Larsen‐Freeman, D., & Schoenemann, T. (2009). Language is a complex adaptive system: Position paper. *Language learning* 59(s1): 1–26. DOI: 10.1111/j.1467-9922.2009.00533.x

Christiansen, M. H., Conway, C. M., & Onnis, L. (2007). Neural responses to structural incongruencies in language and statistical learning point to similar underlying mechanisms. In *Proceedings of the Annual Meeting of the Cognitive Science Society* 29(29): 173–178.

Ettlinger, M., Morgan‐Short, K., Faretta‐Stutenberg, M., & Wong, P. C. (2016). The relationship between artificial and second language learning. *Cognitive science* 40(4): 822-847..

Friederici, A. D., Steinhauer, K., & Pfeifer, E. (2002). Brain signatures of artificial language processing: Evidence challenging the critical period hypothesis. *Proceedings of the National Academy of Sciences* 99(1): 529–534. DOI: 10.1073/pnas.012611199
