# Supplementary material for: Imperfect language learning reduces morphological overspecification: Experimental evidence
Source: PLoS One. 2022 Jan 27;17(1):e0262876. doi: 10.1371/journal.pone.0262876 (PMC8794192; doi:10.1371/journal.pone.0262876)
Supplement: S7 Text — (DOCX) [file pone.0262876.s008.docx]

#### Text S7. Type-token ratio

TTR is defined as the number of distinct words (types) in the language divided by the total number of words (tokens). A word is understood as a sequence of letters delimited by white spaces or other non-word characters. Following Bentz et al. (2015), we do not perform any lemmatization, i.e. *mi*, *mo*, *seg* and *segi* in language 1-0 (see Figure 2) are four different words. In a few languages, we observe what we call "dummy verbs", i.e. verbs that do not map onto any element of the meaning space (any of the three possible events). In language P39-10 (see Appendix S1), for instance, 'round animal' is expressed as *rub io* (while we would expect *rub*). We consider dummy verbs (see language P39-10) separate words (i.e. *rub io* for 'X' counts as two words: *rub* and *io*) in order to prevent the inflation of TTR.

More often, we observe an opposite phenomenon, which we call "empty verbs". In language N1-7, for instance, 'square animal falls apart' is expressed as *fuv* (while we would expect *fuv ro*). In this case, we do not take any measures, that is, we do not introduce a special token "empty string". In other words, if there is an event in the meaning space which is not formally expressed, the empty verb is not counted neither in the number of tokens nor in the number of types. In practice, the presence of empty verbs usually leads to a slightly higher TTR, since the denominator (number of tokens) is smaller compared to a language where the same verb is not empty, while the numerator (number of types) in most cases is not affected. We follow the same principle when calculating TTR of verb stems and endings: if a verb is empty, both the stem and the ending are also empty. In language N30-10, this leads to the necessity of calculating TTR of a verb ending on an empty subcorpus (the meaning 'fall apart' is never expressed), we consider it 0.

References:

Bentz, C., Verkerk, A., Kiela, D., Hill, D., & Buttery, P. (2015). Adaptive Communication: Languages with More Non-Native Speakers Tend to Have Fewer Word Forms. *PLoS ONE*, *10*(6), e0128254. DOI:10.1371/journal.pone.0128254
