# Supplementary material for: Imperfect language learning reduces morphological overspecification: Experimental evidence
Source: PLoS One. 2022 Jan 27;17(1):e0262876. doi: 10.1371/journal.pone.0262876 (PMC8794192; doi:10.1371/journal.pone.0262876)
Supplement: S8 Text — (DOCX) [file pone.0262876.s009.docx]

#### Text S8. Entropy

As a secondary measure, we calculated Shannon entropy for the frequency distribution of wordforms. For every language, the frequency of all wordforms (i.e. sequences of symbols separated by whitespaces) was calculated, and Shannon entropy was calculated for the resulting distribution. Languages may differ in the number of wordforms (sometimes verbs are omitted; sometimes pictures without an event are described by a combination of a noun and a "dummy" verb), no correction for this fact was applied. Change of entropy over time is represented on Figure S4. Using entropy yields approximately the same patterns as using TTR.
