# Supplementary material for: Imperfect language learning reduces morphological overspecification: Experimental evidence
Source: PLoS One. 2022 Jan 27;17(1):e0262876. doi: 10.1371/journal.pone.0262876 (PMC8794192; doi:10.1371/journal.pone.0262876)
Supplement: S9 Text — (DOCX) [file pone.0262876.s010.docx]

#### Text S9. Finer-grained analysis of how meanings are expressed: expressibility

The initial languages had four types of form-meaning pairings (for convenience, we will call them *categories*, implying meaning categories, not morphological categories): noun stem - agent; verb stem - event; noun ending - number of agents; verb ending - agent (redundant agent-marking). We introduce a measure which we dub *expressibility* *of a given category* and which is defined as the proportion of cases where the category is expressed overtly. In this section, we label categories as resp. agent, event, number and agreement.

For agreement, the concept can be easily understood by means of Table 2 in the main text. We ignore the first two rows (as they have no verbal meanings) and then compare pairwise the two cells in the other six rows: are the verbs the same or different? If they are the same, we consider that agreement is not expressed in this particular case. If the verbs are different, we consider that agreement is expressed (no matter what the difference is, i.e. this measure is blind to how regular the system is). The verbs are always different at generation 0, and expressibility equals 6/6 = 1. At generation 10, the verbs are always the same and expressibility equals 0/6 = 0. Note that expressibility does not have to equal either 0 or 1, any intermediate value is possible, and they all have been attested in our languages.

For number, the total number of cases where the category can be expressed is 8 and the criterion would be that the noun form is different. We do pairwise comparisons of row 1 and 2; 3 and 4; 5 and 6; 7 and 8. In language T18-10 (Table 4), for instance, despite its intricate grammar, the expressibility still equals 1.

For agent, we compare noun forms in the two cells of every row from 1 to 8.

For event, the calculation is slightly more complicated, since this category has not two, but three values: ‘fall apart’, ‘grow antlers’, ‘fly’^^[[1]](#footnote-2)^^. We do three pairwise comparisons and then average the three resulting proportions.

Dummy "verbs" (see Text S8) are not taken into account when calculating expressibility, since they are not really expressing a meaning. Empty verbs (see Text S8) are treated as empty strings.

Figure S5 represents changes of the four basic categories over time.

Agreement is the only category where expressibility experiences a noticeable decline.

1. It is also possible to claim that there are four possible meanings, one being null (or just 'be'), but we avoid this solution for simplicity’s sake. [↑](#footnote-ref-2)
