# Supplementary material for: Imperfect language learning reduces morphological overspecification: Experimental evidence
Source: PLoS One. 2022 Jan 27;17(1):e0262876. doi: 10.1371/journal.pone.0262876 (PMC8794192; doi:10.1371/journal.pone.0262876)
Supplement: S3 Fig — (DOCX) [file pone.0262876.s013.docx]

#### Figure S3. Change in transmission fidelity over time


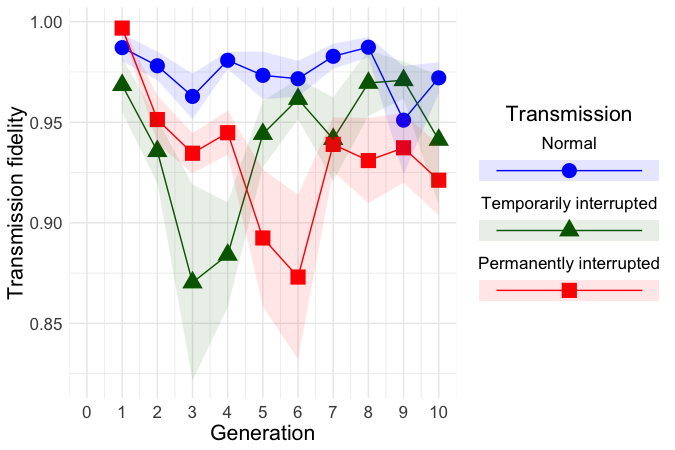


Figure S3. Change in transmission fidelity over time. Shaded bands show standard error.
