# Supplementary material for: Imperfect language learning reduces morphological overspecification: Experimental evidence
Source: PLoS One. 2022 Jan 27;17(1):e0262876. doi: 10.1371/journal.pone.0262876 (PMC8794192; doi:10.1371/journal.pone.0262876)
Supplement: S1 Appendix — (ZIP) [file pone.0262876.s001.zip › Appendix S1/clean_code.zip!/clean_code/end.html]

Спасибо за участие!


Спасибо за участие!
