# Supplementary material for: Imperfect language learning reduces morphological overspecification: Experimental evidence
Source: PLoS One. 2022 Jan 27;17(1):e0262876. doi: 10.1371/journal.pone.0262876 (PMC8794192; doi:10.1371/journal.pone.0262876)
Supplement: S1 Appendix — (ZIP) [file pone.0262876.s001.zip › Appendix S1/clean_code.zip!/clean_code/external_page2.html]

Форма согласия

Подтверждаю, что осведомлен(а) о правилах проведения эксперимента и добровольно соглашаюсь в нем участвовать.

Подтверждаю, что я не лингвист, что я участвую в эксперименте впервые и что русский язык для меня родной.

Ваш пол:
  
Мужской
  
Женский

Ваш возраст:

Начать!
