# Supplementary material for: Imperfect language learning reduces morphological overspecification: Experimental evidence
Source: PLoS One. 2022 Jan 27;17(1):e0262876. doi: 10.1371/journal.pone.0262876 (PMC8794192; doi:10.1371/journal.pone.0262876)
Supplement: S1 Appendix — (ZIP) [file pone.0262876.s001.zip › Appendix S1/clean_code.zip!/clean_code/outro.php]

Спасибо за участие!


Большое спасибо за участие! **Чтобы иметь возможность выиграть приз, запишите все коды, данные ниже, целиком. Эта страница закроется через 3 минуты**. В конце апреля или начале мая часть случайно выбранного кода-победителя будет опубликована на этом сайте. Для получения приза обладателю этого кода нужно будет в течение 30 дней с момента публикации связаться с нами по адресу aleksandrs.berdicevskis@uit.no, указав недостающую часть кода. Кроме того, любой участник сможет узнать, чему был посвящен эксперимент.
